# Spontaneous pneumoperitoneum with duodenal diverticulosis in an elderly patient: a case report

**DOI:** 10.1186/s40792-019-0769-4

**Published:** 2020-01-06

**Authors:** Takeshi Ueda, Tetsuya Tanaka, Takashi Yokoyama, Tomomi Sadamitsu, Suzuka Harada, Atsushi Yoshimura

**Affiliations:** Department of Surgery, Minami-Nara General Medical Center, 8-1 Fukugami, Oyodo, Yoshino, Nara 638-8551 Japan

**Keywords:** Spontaneous pneumoperitoneum, Duodenal diverticulosis, Conservative management

## Abstract

**Background:**

Pneumoperitoneum commonly occurs as a result of a viscus perforation and usually presents with peritoneal signs requiring emergent laparotomy. Spontaneous pneumoperitoneum is a rare condition characterized by intraperitoneal gas with no clear etiology.

**Case presentation:**

We herein report a case in which conservative treatment was achieved for an 83-year-old male patient with spontaneous pneumoperitoneum that probably occurred due to duodenal diverticulosis. He had stable vital signs and slight epigastric discomfort without any other signs of peritonitis. A chest radiograph and computed tomography showed that a large amount of free gas extended into the upper abdominal cavity. Esophagogastroduodenoscopy showed duodenal diverticulosis but no perforation of the upper gastrointestinal tract. He was diagnosed with spontaneous pneumoperitoneum, and conservative treatment was selected. His medical course was uneventful, and pneumoperitoneum disappeared after 6 months.

**Conclusion:**

In the management of spontaneous pneumoperitoneum, recognition of this rare condition and an accurate diagnosis based on symptoms and clinical imaging might contribute to reducing the performance of unnecessary laparotomy. However, in uncertain cases with peritoneal signs, spontaneous pneumoperitoneum is difficult to differentiate from free air resulting from gastrointestinal perforation and emergency exploratory laparotomy should be considered for these patients.

## Background

Pneumoperitoneum is caused by perforation of intraperitoneal organs in more than 90% of patients, which necessitates emergency laparotomy [1, 2]. Spontaneous pneumoperitoneum is a rare condition characterized by intraperitoneal gas without gastrointestinal tract perforation, for which no clear etiology has been identified. If properly diagnosed, some cases of spontaneous pneumoperitoneum can be managed conservatively. However, spontaneous pneumoperitoneum without a known cause is difficult to diagnose.

We herein report a case in which an elderly patient with pneumoperitoneum, but without symptoms of peritonitis, was successfully treated with conservative therapy. We also discuss the possible etiology and clinical issues in the management of this rare condition.

## Case presentation

**Fig. 1:**
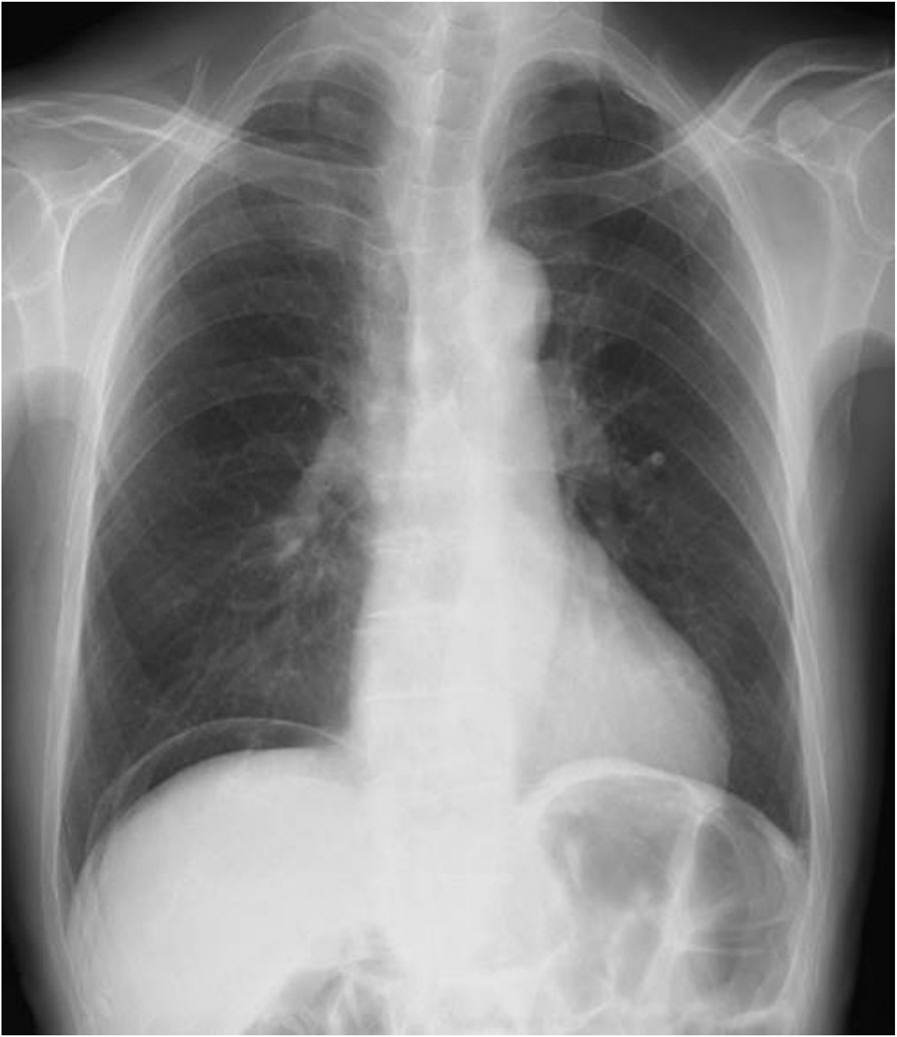
Chest radiograph showing free gas in the right subdiaphragmatic region

**Fig. 2:**
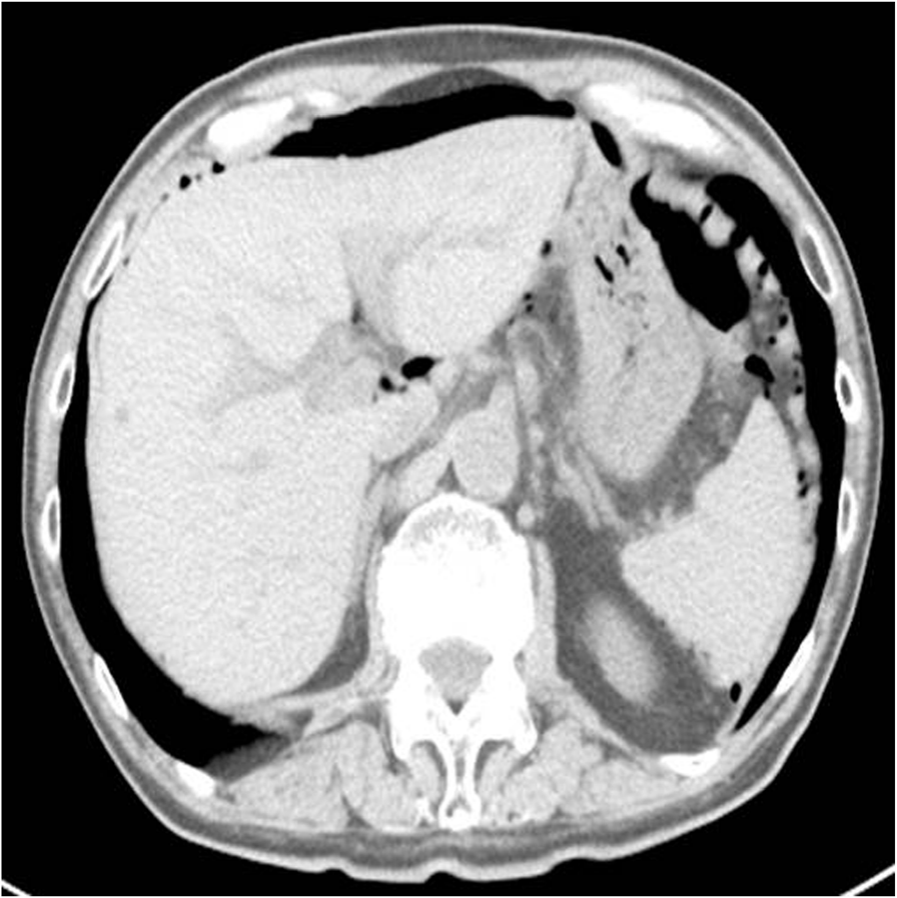
Abdominal CT showing an amount of free gas without intra-abdominal fluid collection in the upper abdominal cavity

An 83-year-old Japanese male patient was referred to our hospital because of a pneumoperitoneum on a chest X-ray film taken by his primary doctor for examination of the cardiac function (Fig. 1). He had a history of treatment for skin cancer, prostate cancer, paroxysmal atrial fibrillation, and laparotomy for appendicitis and bilateral inguinal hernia. The patient took edoxaban tosilate hydrate, bisoprolol fumarate, and famotidine. He did not smoke or consume alcohol. He did not have a fever, dyspepsia, appetite loss, abdominal pain, vomiting, or constipation until the first visit. A physical examination revealed slight epigastric discomfort without any signs of peritonitis. His vital signs were as follows: body temperature, 35.3 °C; pulse rate, 65 beats/min; respiration rate, 18 breaths/min; and blood pressure, 114/71 mmHg. The laboratory data showed a normal white blood cell count of 6300/μl (normal range 3300–8600), a C-reactive protein level of 0.01 mg/dl (normal range < 0.30), and Hb 11.5 g/dl (13.7–16.8). All other laboratory data were within the normal ranges. Abdominal computed tomography (CT) revealed a large amount of free air in the abdomen without fluid collection (Fig. 2).

**Fig. 3:**
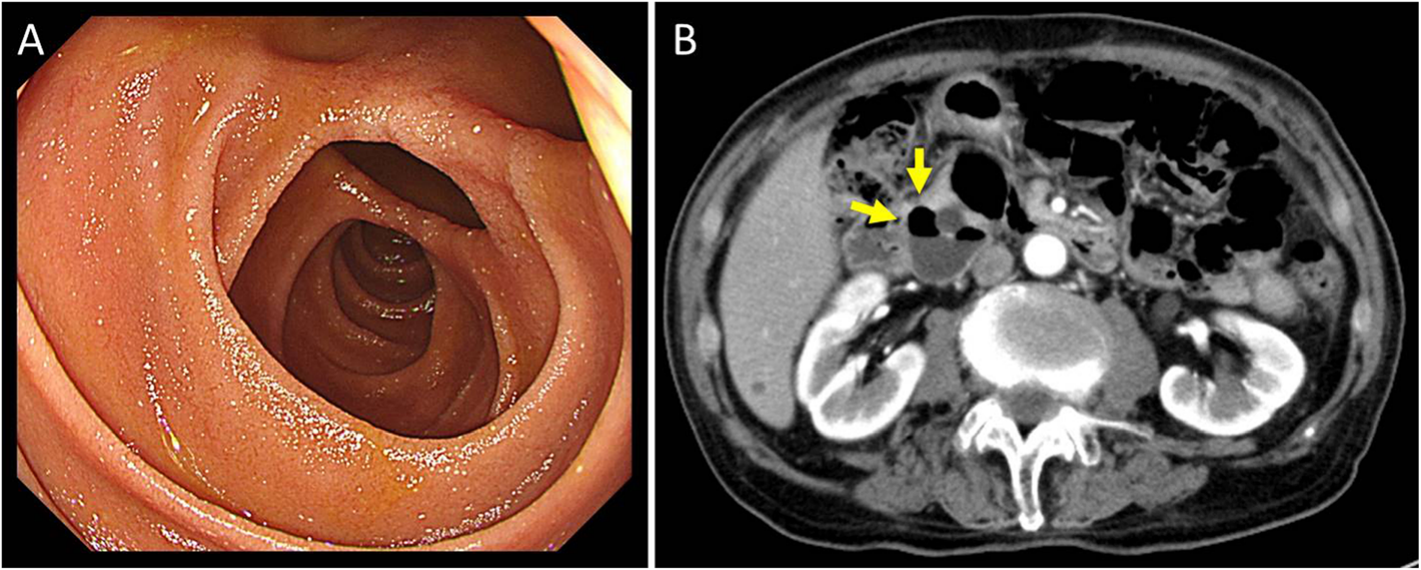
**a** The EGD showed two diverticula in the duodenal second portion. **b** CT showed a diverticulum in the duodenal second portion (arrow)

Based on these findings, the patient was diagnosed with spontaneous pneumoperitoneum. We started conservative treatment, and his general condition remained stable. The clinical course was subsequently uneventful. The laboratory data were normal during the observation period. To explore the cause of pneumoperitoneum, we planned esophagogastroduodenoscopy (EGD) and colonoscopy. He had two large duodenal diverticula and no evidence of perforation in the upper gastrointestinal tract on EGD (Fig. 3). Some sigmoid colonic diverticula were detected by colonoscopy. After 6 months, he had no symptoms, and the intraperitoneal gas spontaneously disappeared.

## Discussion

Pneumoperitoneum occurs as a result of a hollow viscus perforation in more than 90% of patients [1, 2]. Pneumoperitoneum without hollow viscus perforation is a rare phenomenon called spontaneous pneumoperitoneum or non-surgical pneumoperitoneum. Gantt et al. classified the cause of non-surgical pneumoperitoneum as intra-thoracic, abdominal, gynecologic, and iatrogenic [3]. The intra-thoracic causes include mechanical ventilation, cardiopulmonary resuscitation, and pneumothorax, thought to be dissection into the abdominal cavity via transdiaphragmatic or transmediastinal routes. Abdominal causes include pneumatosis cystoides intestinalis (PCI), jejunal diverticulosis, constipation, and colonic pseudo-obstruction [3–9]. Gynecological causes include sexual activity or spa jet-induced pneumoperitoneum [3, 10]. The iatrogenic causes include endoscopic procedures and a postsurgical state [11].

In cases with an abdominal etiology, PCI is the most frequent cause. PCI is characterized by multiple intramural gas-filled cysts in any portion of the gastrointestinal tract. Rupture of these submucosal and subserosal cysts causes pneumoperitoneum. In some pneumoperitoneum cases, diverticular disease and constipation accompany PCI [6, 7]. This may be caused by increased intestinal pressure with or without PCI. In these patients, small amounts of gas may pass through the micropores in the wall of the thinned diverticula or intestinal wall, which can only pass intestinal gas and not intestinal contents. Duodenal diverticulosis, as seen in our patient, can also rarely cause spontaneous pneumoperitoneum. In the present case, the patient had slight epigastric discomfort during the first visit to our hospital, at which time duodenal pressure was considered to be increased for some reason. Given that the duodenal diverticulum was located on the abdominal cavity side, we suspected that intestinal gas had passed through the thinned duodenal diverticulum, thereby resulting in spontaneous pneumoperitoneum.

According to a review of cases of spontaneous pneumoperitoneum, 45 cases (23.0%) in 196 patients required exploratory laparotomy [4]. If abdominal pain and distension are minimal, and peritoneal signs, fever, and leukocytosis are absent, conservative management should be considered for nonsurgical pneumoperitoneum [12]. However, spontaneous pneumoperitoneum poses significant management dilemmas for surgeons, especially when peritoneal signs are present [13, 14]. Although Tani et al. suggested that conservative treatment may be applicable for spontaneous pneumoperitoneum with peritoneal signs, exploratory laparotomy should be considered when signs and symptoms of peritonitis are present [15]. When the site of perforation is not detected during the operation, valvular pneumoperitoneum may also be present due to microperforation of the gastrointestinal tract. Valvular pneumoperitoneum is defined as a valve-like arrangement that permits the escape of air but inhibits the leakage of liquid contents with changes in the pressure within the gastrointestinal tract [16]. Imabun et al. reported a case of recurrent valvular pneumoperitoneum in a patient in whom microperforation of a minute gastric ulcer was detected at autopsy [17]. Thus, when nonsurgical pneumoperitoneum is suspected radiologically but peritoneal signs are present, surgical management may be acceptable. In our institution, we determine the surgical indications for spontaneous pneumoperitoneum based on the presence of peritoneal irradiation sign during physical examination, the inflammatory findings of laboratory tests, and intraabdominal fluid collection of CT. Moreover, operations were uneventful in most adult nonsurgical pneumoperitoneum cases for which surgery is performed.

## Conclusion

With thorough medical history taking, physical examinations, appropriate laboratory tests, and radiological findings, it might be possible to diagnose spontaneous pneumoperitoneum and thereby avoid unnecessary operations. However, in uncertain cases with peritoneal signs, spontaneous pneumoperitoneum should be differentiated from free air resulting from gastrointestinal perforation and emergency exploratory laparotomy should be performed.

## Data Availability

All data supporting this article are included in the published article.

## Abbreviations

CT: Computed tomography
EGD: Esophagogastroduodenoscopy
PCI: Pneumatosis cystoides intestinalis
